# A Case Report of Oral Malignant Melanoma: A Silent Killer

**DOI:** 10.7759/cureus.36671

**Published:** 2023-03-25

**Authors:** Monal M Kukde, Anil U Madurwar, Deepak S Selokar, Obaid Noman

**Affiliations:** 1 Dentistry, Datta Meghe Medical College and Shalinitai Meghe Hospital, Nagpur, IND; 2 Radiodiagnosis, Chandrapur CT Scan and MRI Center, Chandrapur, IND; 3 Community and Family Medicine, Public Health Department, Zilla Parishad, Nagpur, IND; 4 Pathology, Jawaharlal Nehru Medical College, Datta Meghe Institute of Higher Education & Research, Wardha, IND

**Keywords:** tumor, melanocytes, pigmented lesion, melanotic lesion, oral malignant melanoma

## Abstract

Melanoma of the oral cavity is a rare malignant tumor that develops from a malignant melanocytic or de novo from melanocytes within the normal mucosa or skin and appears blue, black, or reddish-brown. Oral mucosal melanoma has a higher proclivity for metastasis and attacks tissue more aggressively than any other malignant tumor in the mouth. Intestinal melanoma of the head and neck is an uncommon type of cancer that should be counted among the deadliest. Malignant melanoma of the oral cavity accounts for only 0.2%-8.0% of all reported melanoma, although accounting for 1.3% of all malignancies. Because most melanotic mucosal lesions are painless at first, the diagnosis is sometimes delayed until the ulcer or growth causes symptoms. Early detection is critical for effective therapy and the only way to improve survival and prognosis in patients with oral malignant melanoma due to its poor prognosis. To avoid oral melanomas, every single colored lesion identified in the oral cavity should be treated with suspicion and adequate inquiry because a colored lesion might expand, and it should be referred for a biopsy to avoid poisoning. This article shows how the oral clinic is important in the diagnosis of oral ulcers and argues that early detection is necessary to enhance patient outcomes.

## Introduction

Melanoma of the oral cavity is a rare malignant tumor that develops from melanocytes within the normal membrane or skin and appears as blue, black, or brown skin. Oral mucosal melanoma has a higher proclivity for metastasizing and attacking tissue than any other malignant tumor in the mouth [1]. Malignant melanoma of the oral cavity arises from the melanocytic cells of the basal layer of the mucosa [2], with an annual incidence of 1.2 per 10 million people [3], accounting for 0.2%-8% of all melanomas [4,5] and 0.5% of all malignant neoplasias of the oral cavity [6,7]. Although the cause is uncertain, it has been associated with pre-existing melanosis, which accounts for 33%-55% of mucosal melanomas of the head and neck, as well as dental irritation [8], cigarette usage, formaldehyde exposure [9], and alcohol consumption. The oral mucosa is only implicated in around 1% of melanomas, and the palate and maxillary gingiva are the most prevalent sites of this tumor.

Melanocytic density varies greatly. Melanocytes are abundant in the skin of the face. Melanocytes are found in the oral mucosa at a rate of one melanocyte per ten basic cells [10]. Major malignant melanoma of the oral cavity is an uncommon neoplasm characterized by severe symptoms caused by malignant mucosal melanocyte alignment [11]. Unlike cutaneous melanoma, which has been associated with sun exposure, the detrimental characteristics of mucosal melanoma are unknown. These melanomas have no obvious link to chemicals, heat, or body fluids (e.g., smoking, drinking, dental hygiene, irritation of teeth or root fragments, dentures, or other oral fluids) when the oral membrane is routinely exposed. Despite the knowledge that an increase in benign intraoral melanocytic (nevi) occurs and is a potential source of oral melanoma, the oral tradition does not have a clear understanding of the sequence of events. Most oral melanomas are assumed to have formed spontaneously around this time.

Male predilection exists, with a male-to-female ratio of roughly 2:1. Oral carcinoma melanoma primarily affects people over the age of 40 and is uncommon in people under the age of 20. Men between the ages of 51 and 60 are more likely to have oral malignant melanoma, whereas women between the ages of 61 and 70 are more likely to develop oral malignant melanoma [12]. Most authors adopt the Western Society of Teachers of Oral Pathology (WESTOP) classification system, which classifies oral malignant melanoma into simple categories based on their histological pattern as (a) Melanoma in situ, identifying the epidermis and epidermis of the melanoma; (b) invasive melanomas, in which neoplasia extends to the connective tissue; and (c) melanoma with a pattern that is intertwined between attack and in situ [13,14].

Atypical melanocytes with a range of morphologies, including a single spindle, transparent plasmacytoid cells, and epikoid cells [13], are generated and entangled between epithelium and connective tissue, as well as connective tissue attack [3]. It also divides oral malignant melanoma into three histological stages, namely, stage I as the main stage, stage II with lymph node metastasis, and phase III with distant metastases [15]. Oral melanoma has a high proclivity for metastasizing to the lymph nodes [13], lungs, liver, brain, and bones.

Because most melanotic mucosal lesions are not painful in the early stages, identification is often delayed until the ulcer or growth leads to symptoms. Oral melanomas develop quietly, with only a few signs, until they have progressed. Because most people do not examine their mouth cavity thoroughly, melanoma progresses until substantial swelling, tooth decay, or bleeding prompts patients to seek medical attention. Wounds ranging in size from 1.0 mm to 1.0 cm or longer are noted. Amelanotic melanoma is a type of oral melanoma that presents as a white, red, or reddish spot in the mouth. Pigmentation deficiency adds to inaccurate clinical and histological diagnoses.

Oral melanomas are typically macular; however, they can also include pedunculated ulcers. Pain, ulceration, and bleeding are common symptoms of oral melanomas. In mucosal lesions, white specks are occasionally detected, and erythema emerges when the lesions are irritated. Moreover, pigmentation ranges from dark brown to dark blue to black. The palate and maxillary gingiva are damaged in roughly 80% of patients, but mucous membranes, mandibular gingiva, and tongue ulcers are also noted [16].

Unless the primary tumor is large, a lump in the neck may indicate regional metastasis, which is uncommon. Oral melanoma of any mucosal surface is yet to be identified. Skin lesions, on the other hand, are related to people with blue skin and eyes who have had a history of sunburn, and their prevalence has risen considerably. The prognosis of oral melanoma on the palate is unknown. There is no link between dentures, chemical or physical abuse, or cigarette use. Blue nevi, which are melanocytic lesions, are more frequent in the palate. There have been no known side effects of oral blue nevi. Addison disease, ephelides (Freckles), blue nevi, Kaposi sarcoma, and oral nevi are all different diagnoses. Oral melanoma is frequently ignored or misdiagnosed as a white-skinned procedure until it progresses substantially. Given the anatomic complexity and regional lymphatic drainage, an intensive surgical technique is required. Alternative treatments for oral melanomas include total wound surgery with a protective edge, as well as radiotherapy, chemotherapy, or a combination of the two [17,18].

The prognosis for oral melanoma is poor, with a five-year survival rate of around 10% to 25%. Life expectancy is fewer than two years on average [19]. Mouth melanomas are similarly aggressive as other oral cancers or cutaneous melanomas, spreading and metastasizing swiftly. Early detection and diagnosis are critical for a better prognosis and survival rate in oral cancer. Any single lesion identified in the oral cavity should be treated with suspicion to prevent oral melanomas, and a complete biopsy investigation should be undertaken to eliminate the lesion. Early detection and treatment increase prediction greatly [20]. This paper illustrates how the oral clinic plays an important role in the diagnosis of oral ulcers and emphasizes the importance of treating any colored sores in the mouth with suspicion and conducting appropriate investigations (radiology, hematology, biopsy, etc.) to prevent negative outcomes.

## Case presentation

A 70-year-old female patient, a farmer by occupation with a fair complexion, medium height, and a built-in scale, visited the outpatient department with the major complaint of a non-tender growth in the right back area of the maxilla for one month.

**Figure 1:**
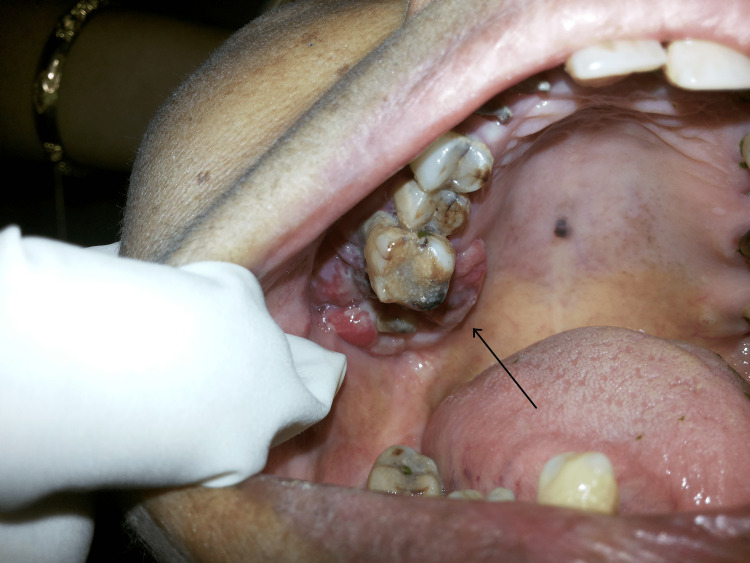
Black exophytic growth behind the right maxillary alveolus noted on the initial visit.

Poor nutrition and loose teeth were linked to rapid growth. The patient stated that no one in her family had experienced similar issues. Her other complaints were less significant. A physical examination revealed no significant results. The patient was known for consuming betel nuts and putting them in the oral cavity and chewing for long hours. A painless black exophytic growth behind the right maxillary alveolus posteriorly spanning the size of the gingiva between the teeth approximately 4 × 3 cm on the buccal and palatal side extending across the 14 to 17 involving gingival sulcus and alveolus was discovered on examination of the oral cavity, which may have originated from the gingival tissue (Figure 1). The front of the face, from the mesial area of the 14th gingiva to the 17th gingiva in the back, was smooth and uneven. The wound was extending to the top of the mouth.

It extended to the palatal mucosa, with 15 and 16 indicating mobility. The palpatory effects revealed a wound with a hard consistency and smooth texture. There was no bleeding in the growth. The lymph nodes in the region were undetectable. After a thorough inspection of the wound, no more large sites of the lesion were discovered. Based on the clinical symptoms, a temporary diagnosis of oral melanoma involving the right maxillary alveolus was made. Kaposi’s sarcoma, melanotic macule, and any vascular-related illnesses were also suspected diagnoses. Basic investigations were undertaken to rule out the diagnosis, including hematological parameters, an orthopantomogram (OPG), and a CT scan. The results of the hematological investigation revealed no significant findings, indicating that the lesion was not hematological. The OPG revealed a soft tissue shadow in the right arch at the 18, 17, and 16 areas, as well as a thick mucous membrane on the lower border of the maxillary sinus.

**Figure 2:**
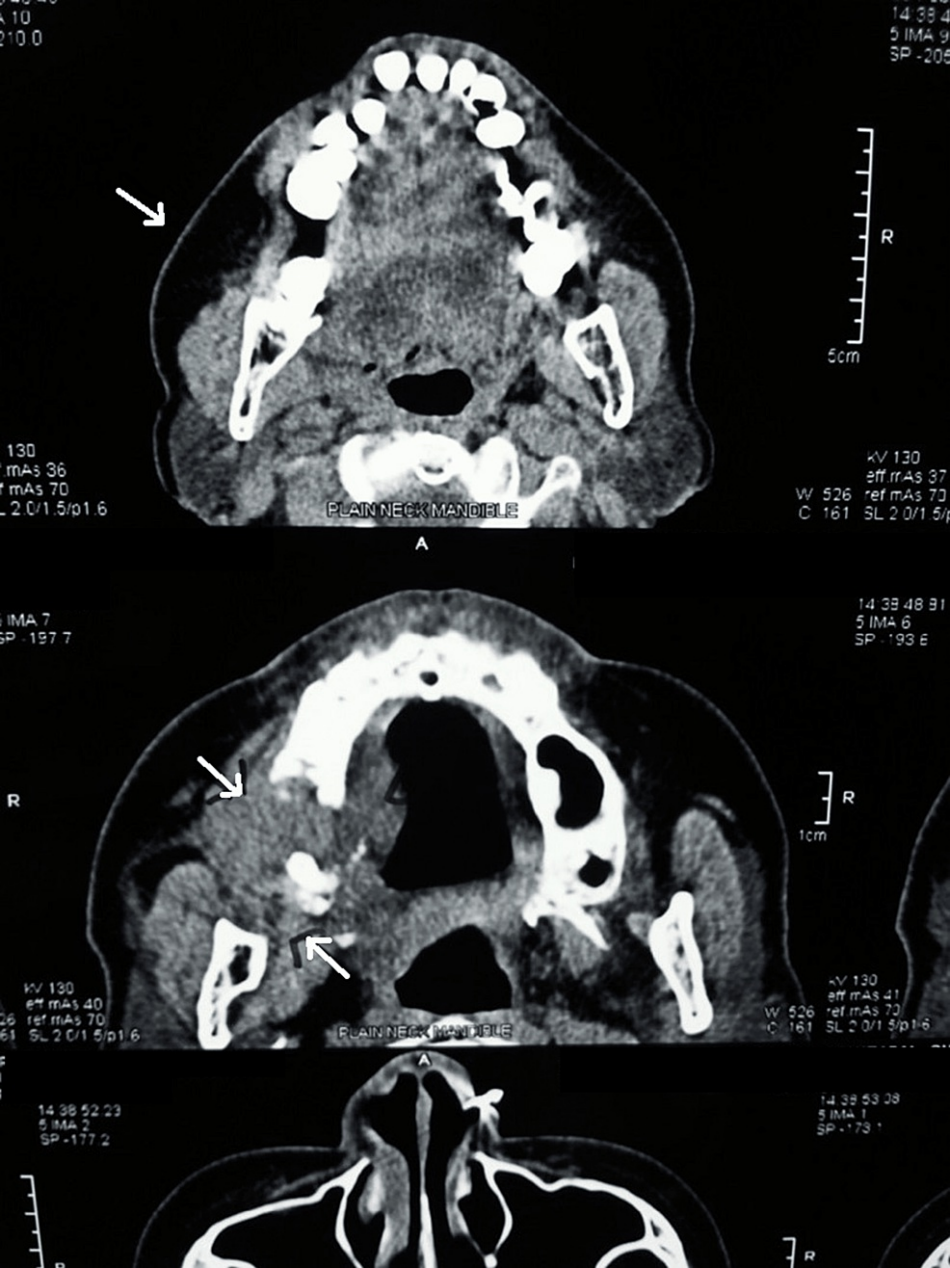
CT scan showing hypodense soft tissue density on the upper right alveolar arch.

**Figure 3:**
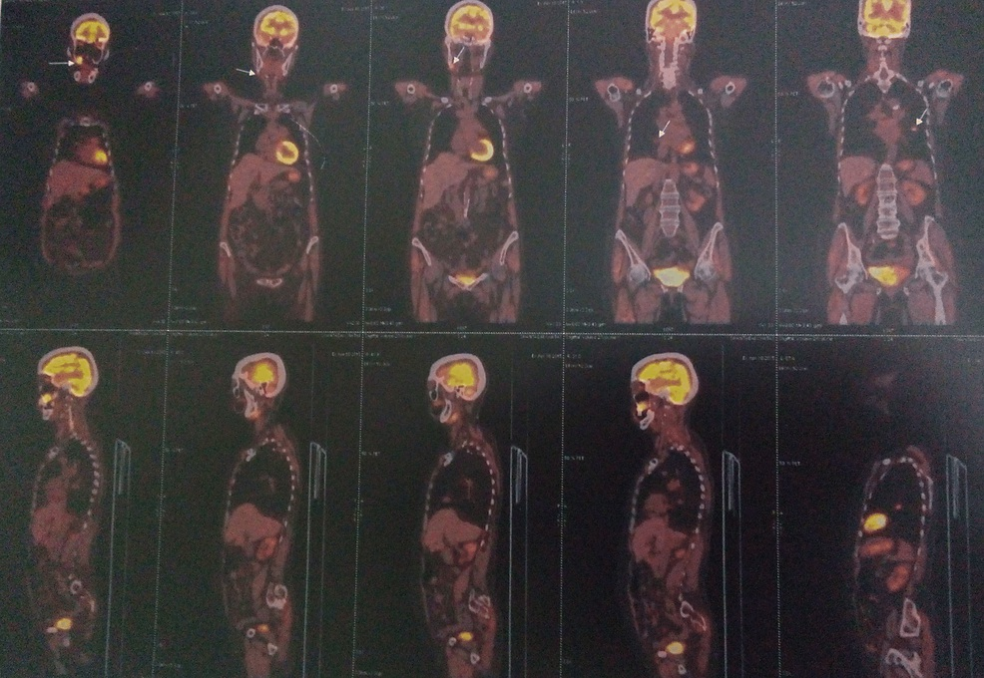
Positron emission tomography scan showing metastasis in the lungs.

A CT scan revealed hypodense soft tissue density that developed into a massive lesion that appeared to cover the upper right alveolar arch. The alveolus was eroded. The right maxillary sinus was involved, and it produced aberrant bone degeneration (Figure 2). The right gingiva buccal sulcus was affected by weight reduction, which increased the risk of neoplastic etiology. Positron emission tomography (PET) scans were obtained to evaluate the metastasis of the lesion revealing fluorodeoxyglucose (FDG) lesion with a right maxillary alveolar region, adjacent buccogingival sulcus, and palate. The FDG-avid lesion was also observed in the right lung. There was evidence of a low FDG-avid small lesion observed in the medial segment of the lower right lung parenchyma (Figure 3).

**Figure 4:**
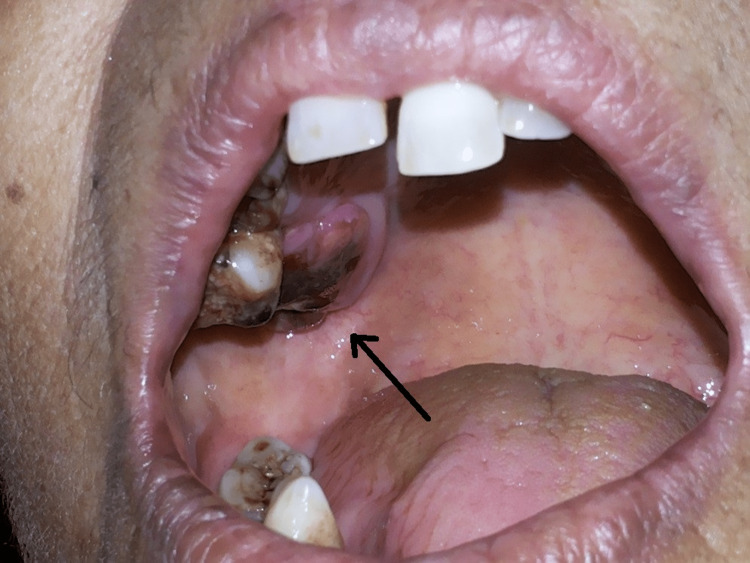
Follow-up visit after three months showing the increased extent of the lesion.

To make a correct diagnosis, an incisional biopsy was performed under local anesthesia after clinical observations and radiographic features. A biopsy sample was delivered to the Department of Pathology, TATA Memorial Hospital, Mumbai. Immunohistochemistry was positive for Melan-A, and CD30, CD23, CD3, and CD20 Mib-1 were colorless in 60% of cases. Malignant melanoma was the most recent diagnosis. The patient presented after three months and the size and extent of the lesion had increased (Figure 4). The patient refused to follow any traditional treatment procedure and lived for nearly two years after the initial visit.

## Discussion

Mucosal melanoma of the oral cavity is a rare malignant illness that affects between 0.2% and 8% of all melanoma cases [21]. The most prevalent lesion is detected in the pelvis (47.4%), followed by the upper maxillary mucosa (27.8%), confirming what Meleti et al. described [22]. In addition, 41 of the 119 ulcers (34.4%) in the pelvis, followed by the upper maxillary mucosa, as reported by Doval et al. [23] in 14 patients, 11 of whom had ulcers on the palate.

Lopez-Graniel et al. discovered that 66.6% of patients have a diameter of 4 cm or greater [24]. Although most melanomas show on the skin, they can develop elsewhere in the melanocyte. Damage from ultraviolet light is thought to be a key source of melanomas, as evidenced by the fact that melanoma proliferation rises in people as they approach the equator, even if prolonged sun exposure is less visible than other skin diseases such as basal and squamous cell carcinoma. Sun exposure is not associated with intraoral mucosal lesions [25].

Oral malignant melanoma has a wide range of morphologic characteristics, process development, and biological behavior [26]. It is now widely understood that mouth cancer is a type of cancer. Melanoma is a highly aggressive tumor, with features such as late identification, poor resectability, and early spread contributing to its aggressiveness. This not only reduces the five-year survival rate to 10%-25% but also impacts the prognosis. Chemotherapy is highly resistant to melanoma; however, other treatments can be used in conjunction with it.

Table 1 summarizes the clinical and Table 2 summarizes the radiological (Table 2) features of the patient’s case, including the description of the exophytic growth and the CT and PET scan findings. The tables provide a concise overview of the key features of the case, which can be helpful for clinicians in making a diagnosis and determining the appropriate course of treatment.

**Table 1:** Clinical features.

Clinical feature	Description
Location	Behind the right maxillary alveolus
Size	Approximately 4 × 3 cm
Color	Painless black exophytic growth
Extent	Spans the size of the gingiva between the teeth
Side	On the buccal and palatal side
Involvement	Extends across 14 to 17 involving gingival sulcus and alveolus
Mobility	15 and 16 indicating mobility
Palpation	Wound with hard consistency and smooth texture on palpation
Palatal extension	Extends to the palatal mucosa

**Table 2:** Radiological features. FDG: fluorodeoxyglucose; PET: positron emission tomography

Radiological feature	Description
Imaging technique	CT and PET scans
Soft tissue density	Hypodense soft tissue density on the CT scan
Lesion size	Massive lesion covering the upper right alveolar arch
Maxillary sinus involvement	Erosion of the right maxillary sinus
Bone degeneration	Aberrant bone degeneration in the right maxillary sinus
Buccal sulcus	Weight reduction affecting the right gingival buccal sulcus
PET scan findings	FDG-avid lesion in the right maxillary alveolar region, adjacent buccogingival sulcus, and palate
Lung involvement	FDG-avid lesion observed in the right lung
Lung lesion	Evidence of a low FDG-avid small lesion in the medial segment of the lower right lung parenchyma

The differential diagnosis list for oral melanosis includes various conditions that share similar physical characteristics such as oral melanotic macule, smoking-related melanosis, medication-induced melanosis caused by antimalarial drugs and minocycline, melanoplakia, pituitary-based Cushing syndrome, postinflammatory pigmentation, melanoacanthoma, melanocytic nevi of the oral mucosa, blue nevi, nevi of Spitz, Addison’s disease, amalgam tattoo, Kaposi’s sarcoma, physiologic pigmentation, pigmentation related to the use of heavy metals, and many more. It is important not to overlook any of these potential diagnoses [27]. Oral melanomas can be primary or secondary to other cancers. The etiopathogenesis of many diseases is unknown. Precursor lesions, such as previous melanosis or atypical melanocytic hyperplasia, may signify a proliferative stage before carcinogenesis [3]. Tanaka et al. [28], discovered 18 cases of metastases out of 35 cases evaluated in their investigation (52.43%) Other probable etiological causes include mechanical damage, such as tooth irritation, tobacco usage, formaldehyde exposure, and alcohol consumption [21].

## Conclusions

In this study, a steady increase in melanoma in the oral cavity was detected, with the maximum increase at 16 years of life, and is generally seen in the palate. Primary oral mucosal melanomas are physiologically invasive and extremely rare. Oral melanomas resemble other pigmented oral tumors in appearance. Any colored sore in the oral cavity should be treated with caution, and any colored sore that can be diagnosed clinically should be evaluated. Malignant melanoma should be included in a separate diagnosis of pigmented lesions by the dentist because early detection and action can lead to a better prognosis.
